# (*E*)-4-Methyl-*N*′-[(4-oxo-4*H*-chromen-3-yl)methyl­idene]benzohydrazide

**DOI:** 10.1107/S1600536814008113

**Published:** 2014-04-16

**Authors:** Yoshinobu Ishikawa, Kohzoh Watanabe

**Affiliations:** a School of Pharmaceutical Sciences, University of Shizuoka, 52-1 Yada, Suruga-ku, Shizuoka 422-8526, Japan

## Abstract

In the title chromone-tethered benzohydrazide derivative, C_18_H_14_N_2_O_3_, the 4*H*-chromen-4-one and the –CH=N–NH–CO– units are each essentially planar, with the largest deviations from thei planes being 0.052 (2) and 0.003 (2) Å, respectively. The dihedral angles between the 4*H*-chromen-4-one and the –CH=N–NH–CO– units, the 4*H*-chromen-4-one unit and the benzene ring of the 4-tolyl group, and the benzene ring of the 4-tolyl group and the –CH=N–NH–CO– unit are 8.09 (7), 9.94 (5) and 17.97 (8)°, respectively. In the crystal, the mol­ecules form two types of centrosymmetric dimers: one by N—H⋯O hydrogen bonds and the other by π–π stacking inter­actions between the 4*H*-chromen-4-one unit and the 4-tolyl group [centroid–centroid distance = 3.641 (5) Å]. These dimers form one-dimensional assemblies extending along the *a-*axis direction. Additional π–π stacking inter­actions between two 4*H*-chromen-4-one units [centroid–centroid distance = 3.591 (5) Å] and two 4-tolyl groups [centroid–centroid distance = 3.792 (5) Å] organize the mol­ecules into a three-dimensional network.

## Related literature   

For the biological activity of related compounds, see: Khan *et al.* (2009 ▶); Tu *et al.* (2013 ▶). For a related structure, see: Ishikawa & Watanabe (2014 ▶).
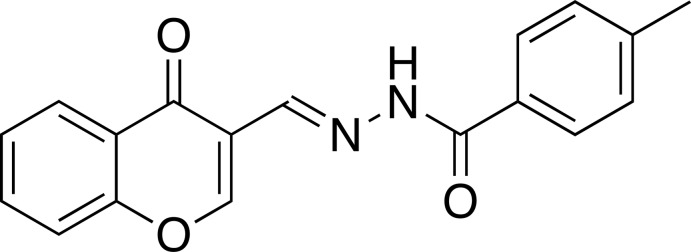


## Experimental   

### 

#### Crystal data  

C_18_H_14_N_2_O_3_
*M* *_r_* = 306.32
Triclinic, 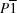

*a* = 7.759 (10) Å
*b* = 8.543 (7) Å
*c* = 11.047 (15) Å
α = 103.55 (11)°
β = 95.53 (12)°
γ = 94.60 (9)°
*V* = 704.6 (15) Å^3^
*Z* = 2
Mo *K*α radiation
μ = 0.10 mm^−1^
*T* = 100 K
0.50 × 0.40 × 0.15 mm

#### Data collection  

Rigaku AFC-7R diffractometer
3953 measured reflections
3235 independent reflections
2434 reflections with *F* ^2^ > 2σ(*F* ^2^)
*R*_int_ = 0.016
3 standard reflections every 150 reflections intensity decay: 1.0%

#### Refinement  

*R*[*F* ^2^ > 2σ(*F* ^2^)] = 0.054
*wR*(*F* ^2^) = 0.187
*S* = 1.09
3235 reflections
209 parameters
H-atom parameters constrained
Δρ_max_ = 0.55 e Å^−3^
Δρ_min_ = −0.43 e Å^−3^

### 

Data collection: *WinAFC Diffractometer Control Software* (Rigaku, 1999 ▶); cell refinement: *WinAFC Diffractometer Control Software*; data reduction: *WinAFC Diffractometer Control Software*; program(s) used to solve structure: *SIR2008* (Burla *et al.*, 2007 ▶); program(s) used to refine structure: *SHELXL97* (Sheldrick, 2008 ▶); molecular graphics: *CrystalStructure* (Rigaku, 2010 ▶); software used to prepare material for publication: *CrystalStructure*.

## Supplementary Material

Crystal structure: contains datablock(s) General, I. DOI: 10.1107/S1600536814008113/gk2609sup1.cif

Structure factors: contains datablock(s) I. DOI: 10.1107/S1600536814008113/gk2609Isup2.hkl

Supporting information file. DOI: 10.1107/S1600536814008113/gk2609Isup3.cml

CCDC reference: 996675

Additional supporting information:  crystallographic information; 3D view; checkCIF report

## Figures and Tables

**Table 1:** Hydrogen-bond geometry (Å, °)

*D*—H⋯*A*	*D*—H	H⋯*A*	*D*⋯*A*	*D*—H⋯*A*
N2—H2⋯O2^i^	0.88	2.22	3.012 (4)	151

Symmetry code: (i) 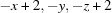.

**Fig. 1.**
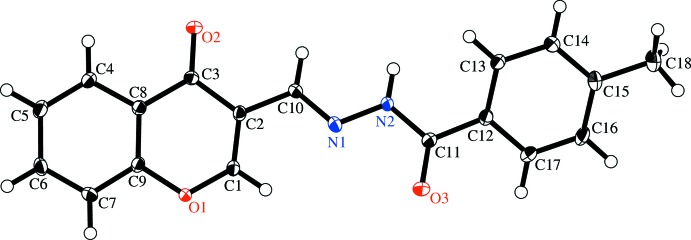
The molecular structure of the title compound, with displacement ellipsoids drawn at the 50% probability level. Hydrogen atoms are shown as small spheres of arbitrary radius.

**Fig. 2.**
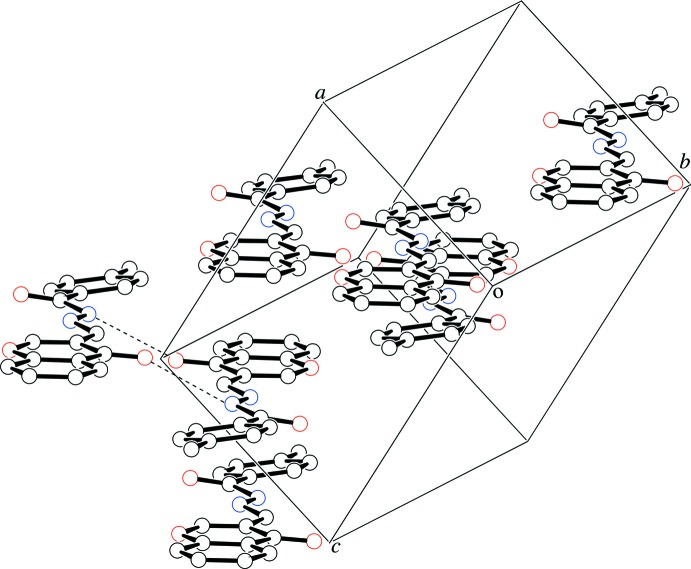
A crystal packing view of the title compound. Intermolecular N–H···O hydrogen bonds are represented by dashed lines. Hydrogen atoms are omitted for clarity.

## supplementary crystallographic information

### 1. Comment

Schiff bases derived from 3-formyl chromones have attracted much attention due to their biological functions such as enzyme inhibition (Khan *et al.*, 2009; Tu *et al.*, 2013). We herein report the crystal structure of the title compound, which was prepared by the condensation reaction of 3-formylchromone with 4-methylbenzoylhydrazide in ethanol. The structure (Figure 1) shows that the atoms of both the 4*H*-chromen-4-one and the –CH=N–NH–CO– segments are essentially coplanar, and the largest deviations are 0.052 (2) for C2 and 0.003 (2) Å for C11, respectively. The dihedral angles between the 4*H*-chromen-4-one segment and the –CH=N–NH–CO– segment, the 4*H*-chromen-4-one segment and the benzene ring of the 4-methylbenzene segment, and the benzene ring of the 4-methylbenzene segment and the –CH=N–NH–CO– segment are 8.09 (7), 9.94 (5), and 17.97 (8)°, respectively. In the crystal, the molecules related by inversion center (symmetry code: –*x* + 2, –*y*, –*z* + 2) are linked by N–H···O hydrogen bonds to form a dimer. There is also an extensive sytem of π···π stacking interactions between inversion related molecules involving both the 4*H*-chromen-4-one unit and the 4-tolyl group.

### 2. Experimental

4-Methylbenzoylhydrazide (1.00 mmol), 3-formylchromone (1.00 mmol), and a few drops of acetic acid were dissolved in 25 ml of ethanol, and the mixture was stirred for 6 h at room temperature. The precipitate was collected, washed with ethanol, and dried *in vacuo* (yield 55.0%). ^1^H NMR (400 MHz, CDCl_3_): *δ* = 3.87 (s, 3H), 6.96 (d, 2H, *J* = 8.3 Hz), 7.41 (t, 1H, *J* = 7.6 Hz), 7.53 (d, 1H, *J* = 8.3 Hz), 7.72 (t, 1H, *J* = 7.6 Hz), 7.90 (d, 2H, *J* = 7.9 Hz), 8.13 (d, 1H, *J* = 8.3 Hz), 8.56 (s, 1H), 8.84 (s, 1H), 9.77 (s, 1H). DART-MS calcd for [C_18_H_14_N_2_O_3_ + H^+^]: 307.321, found 307.156. Single crystals suitable for X-ray diffraction were obtained by slow evaporation of an acetonitrile solution of the title compound at room temperature.

### 3. Refinement

The C(*sp*^2^)- and N(*sp*^2^)-bound hydrogen atoms were placed in geometrically determined positions [C–H 0.95 Å, *U*_iso_(H) = 1.2*U*_eq_(C), N–H 0.88 Å, *U*_iso_(H) = 1.2*U*_eq_(N)], and refined using a riding model. Hydrogen atoms of methyl group were found in a difference Fourier map, and a rotating group model was applied with the distance constraint [C–H = 0.98 Å, *U*_iso_(H) = 1.2*U*_eq_(C)].

### Crystal data

**Table:**

C_18_H_14_N_2_O_3_	*Z* = 2
*M**_r_* = 306.32	*F*(000) = 320.00
Triclinic, *P*1	*D*_x_ = 1.444 Mg m^−^^3^
Hall symbol: -P 1	Mo *K*α radiation, λ = 0.71069 Å
*a* = 7.759 (10) Å	Cell parameters from 25 reflections
*b* = 8.543 (7) Å	θ = 15.3–17.5°
*c* = 11.047 (15) Å	µ = 0.10 mm^−^^1^
α = 103.55 (11)°	*T* = 100 K
β = 95.53 (12)°	Plate, colorless
γ = 94.60 (9)°	0.50 × 0.40 × 0.15 mm
*V* = 704.6 (15) Å^3^	

### Data collection

**Table:**

Rigaku AFC-7R diffractometer	θ_max_ = 27.5°
ω–2θ scans	*h* = −10→5
3953 measured reflections	*k* = −11→11
3235 independent reflections	*l* = −14→14
2434 reflections with *F*^2^ > 2σ(*F*^2^)	3 standard reflections every 150 reflections
*R*_int_ = 0.016	intensity decay: 1.0%

### Refinement

**Table:**

Refinement on *F*^2^	Secondary atom site location: difference Fourier map
*R*[*F*^2^ > 2σ(*F*^2^)] = 0.054	Hydrogen site location: inferred from neighbouring sites
*wR*(*F*^2^) = 0.187	H-atom parameters constrained
*S* = 1.09	*w* = 1/[σ^2^(*F*_o_^2^) + (0.1269*P*)^2^ + 0.0754*P*] where *P* = (*F*_o_^2^ + 2*F*_c_^2^)/3
3235 reflections	(Δ/σ)_max_ < 0.001
209 parameters	Δρ_max_ = 0.55 e Å^−^^3^
0 restraints	Δρ_min_ = −0.43 e Å^−^^3^
Primary atom site location: structure-invariant direct methods	

### Special details

**Table:**

Refinement. Refinement was performed using all reflections. The weighted *R*-factor (*wR*) and goodness of fit (*S*) are based on *F*^2^. *R*-factor (gt) are based on *F*. The threshold expression of *F*^2^ > 2.0 σ(*F*^2^) is used only for calculating *R*-factor (gt).

### Fractional atomic coordinates and isotropic or equivalent isotropic displacement parameters (Å^2^)

**Table:**

	*x*	*y*	*z*	*U*_iso_*/*U*_eq_	
O1	0.70112 (16)	0.52823 (15)	1.02907 (12)	0.0164 (4)	
O2	1.05971 (17)	0.25136 (15)	1.14769 (13)	0.0185 (4)	
O3	0.33072 (18)	−0.08140 (17)	0.74095 (14)	0.0243 (4)	
N1	0.6201 (2)	0.04137 (19)	0.89360 (15)	0.0162 (4)	
N2	0.5919 (2)	−0.12076 (18)	0.83493 (15)	0.0153 (4)	
C1	0.6869 (3)	0.3661 (2)	0.98690 (17)	0.0152 (4)	
C2	0.7963 (3)	0.2663 (2)	1.02346 (16)	0.0130 (4)	
C3	0.9490 (3)	0.3343 (2)	1.11510 (16)	0.0134 (4)	
C4	1.0907 (3)	0.5952 (3)	1.25872 (17)	0.0164 (4)	
C5	1.0995 (3)	0.7613 (3)	1.30115 (18)	0.0193 (5)	
C6	0.9761 (3)	0.8487 (3)	1.25160 (18)	0.0180 (4)	
C7	0.8436 (3)	0.7679 (3)	1.16113 (18)	0.0162 (4)	
C8	0.9585 (3)	0.5112 (2)	1.16508 (16)	0.0131 (4)	
C9	0.8369 (3)	0.6008 (2)	1.11925 (17)	0.0137 (4)	
C10	0.7627 (3)	0.0913 (2)	0.96497 (16)	0.0143 (4)	
C11	0.4381 (3)	−0.1734 (3)	0.75811 (17)	0.0155 (4)	
C12	0.4090 (3)	−0.3499 (2)	0.69518 (17)	0.0145 (4)	
C13	0.4972 (3)	−0.4671 (3)	0.73642 (17)	0.0164 (4)	
C14	0.4579 (3)	−0.6300 (3)	0.67544 (18)	0.0180 (4)	
C15	0.3306 (3)	−0.6795 (3)	0.57241 (17)	0.0177 (4)	
C16	0.2422 (3)	−0.5614 (3)	0.53282 (18)	0.0214 (5)	
C17	0.2796 (3)	−0.3990 (3)	0.59301 (18)	0.0204 (5)	
C18	0.2909 (3)	−0.8561 (3)	0.50609 (19)	0.0257 (5)	
H1	0.5911	0.3175	0.9258	0.0182*	
H2	0.6689	−0.1878	0.8461	0.0183*	
H4	1.1746	0.5375	1.2931	0.0197*	
H5	1.1898	0.8171	1.3644	0.0231*	
H6	0.9841	0.9632	1.2805	0.0216*	
H7	0.7583	0.8255	1.1280	0.0195*	
H10	0.8440	0.0186	0.9799	0.0172*	
H13	0.5846	−0.4357	0.8065	0.0196*	
H14	0.5188	−0.7087	0.7046	0.0216*	
H16	0.1544	−0.5930	0.4631	0.0256*	
H17	0.2169	−0.3207	0.5647	0.0244*	
H18A	0.3058	−0.9223	0.5671	0.0309*	
H18B	0.3705	−0.8846	0.4426	0.0309*	
H18C	0.1706	−0.8764	0.4654	0.0309*	

### Atomic displacement parameters (Å^2^)

**Table:**

	*U*^11^	*U*^22^	*U*^33^	*U*^12^	*U*^13^	*U*^23^
O1	0.0150 (7)	0.0108 (7)	0.0199 (7)	0.0035 (5)	−0.0056 (5)	−0.0008 (5)
O2	0.0158 (7)	0.0155 (7)	0.0227 (7)	0.0054 (5)	−0.0039 (5)	0.0028 (6)
O3	0.0196 (8)	0.0179 (7)	0.0312 (8)	0.0074 (6)	−0.0074 (6)	0.0003 (6)
N1	0.0163 (8)	0.0113 (8)	0.0191 (8)	0.0023 (6)	0.0004 (6)	0.0002 (6)
N2	0.0127 (8)	0.0110 (8)	0.0189 (8)	0.0026 (6)	−0.0023 (6)	−0.0015 (6)
C1	0.0141 (9)	0.0125 (9)	0.0163 (9)	0.0011 (7)	−0.0013 (7)	−0.0006 (7)
C2	0.0113 (9)	0.0121 (9)	0.0144 (9)	−0.0007 (7)	0.0010 (7)	0.0016 (7)
C3	0.0121 (9)	0.0135 (9)	0.0144 (9)	0.0030 (7)	0.0014 (7)	0.0023 (7)
C4	0.0137 (9)	0.0170 (9)	0.0166 (9)	0.0014 (7)	−0.0017 (7)	0.0017 (7)
C5	0.0171 (9)	0.0193 (10)	0.0174 (9)	0.0003 (7)	−0.0030 (7)	−0.0009 (8)
C6	0.0189 (10)	0.0123 (9)	0.0198 (9)	0.0012 (7)	0.0017 (8)	−0.0018 (7)
C7	0.0155 (9)	0.0133 (9)	0.0198 (9)	0.0047 (7)	0.0018 (7)	0.0029 (7)
C8	0.0119 (9)	0.0135 (9)	0.0131 (9)	0.0017 (7)	0.0018 (7)	0.0012 (7)
C9	0.0118 (9)	0.0141 (9)	0.0136 (8)	−0.0000 (7)	0.0001 (7)	0.0012 (7)
C10	0.0138 (9)	0.0117 (9)	0.0164 (9)	0.0031 (7)	0.0006 (7)	0.0013 (7)
C11	0.0134 (9)	0.0155 (9)	0.0163 (9)	0.0021 (7)	−0.0008 (7)	0.0022 (7)
C12	0.0134 (9)	0.0148 (9)	0.0140 (9)	0.0004 (7)	0.0007 (7)	0.0014 (7)
C13	0.0143 (9)	0.0166 (9)	0.0160 (9)	0.0017 (7)	−0.0017 (7)	0.0010 (7)
C14	0.0181 (10)	0.0155 (9)	0.0191 (10)	0.0038 (7)	−0.0000 (8)	0.0016 (7)
C15	0.0200 (10)	0.0149 (9)	0.0159 (9)	−0.0013 (7)	0.0035 (7)	−0.0005 (7)
C16	0.0242 (11)	0.0204 (10)	0.0152 (9)	−0.0024 (8)	−0.0075 (8)	0.0014 (8)
C17	0.0199 (10)	0.0194 (10)	0.0198 (10)	0.0009 (8)	−0.0067 (8)	0.0051 (8)
C18	0.0316 (12)	0.0194 (10)	0.0209 (10)	0.0012 (8)	−0.0013 (9)	−0.0034 (8)

### Geometric parameters (Å, º)

**Table:**

O1—C1	1.345 (3)	C13—C14	1.391 (3)
O1—C9	1.375 (3)	C14—C15	1.393 (4)
O2—C3	1.236 (3)	C15—C16	1.393 (4)
O3—C11	1.221 (3)	C15—C18	1.508 (3)
N1—N2	1.375 (3)	C16—C17	1.385 (3)
N1—C10	1.275 (3)	N2—H2	0.880
N2—C11	1.374 (3)	C1—H1	0.950
C1—C2	1.350 (3)	C4—H4	0.950
C2—C3	1.466 (4)	C5—H5	0.950
C2—C10	1.474 (3)	C6—H6	0.950
C3—C8	1.476 (3)	C7—H7	0.950
C4—C5	1.380 (3)	C10—H10	0.950
C4—C8	1.403 (4)	C13—H13	0.950
C5—C6	1.407 (4)	C14—H14	0.950
C6—C7	1.377 (4)	C16—H16	0.950
C7—C9	1.389 (3)	C17—H17	0.950
C8—C9	1.392 (3)	C18—H18A	0.980
C11—C12	1.494 (3)	C18—H18B	0.980
C12—C13	1.394 (3)	C18—H18C	0.980
C12—C17	1.397 (4)		
			
O1···C3	2.871 (4)	C5···H14^v^	3.0350
O2···C1	3.573 (5)	C5···H17^vii^	3.2163
O2···C4	2.887 (4)	C5···H18B^ix^	3.4854
O2···C10	2.926 (5)	C5···H18C^ix^	3.1753
O3···N1	2.655 (5)	C5···H18C^iv^	3.4913
O3···C17	2.796 (4)	C6···H10^ii^	3.4010
N1···C1	2.712 (4)	C6···H18A^iv^	3.1090
N2···C13	2.910 (4)	C6···H18C^ix^	3.0855
C1···C7	3.571 (5)	C6···H18C^iv^	3.3918
C1···C8	2.737 (5)	C7···H1^i^	3.3884
C2···C9	2.786 (4)	C7···H10^iii^	3.2563
C4···C7	2.796 (4)	C7···H14^iv^	3.3602
C5···C9	2.746 (5)	C7···H18A^iv^	3.3403
C6···C8	2.805 (4)	C9···H1^i^	3.4609
C10···C11	3.498 (6)	C9···H14^iv^	3.5858
C12···C15	2.814 (4)	C10···H6^ii^	3.4658
C13···C16	2.768 (5)	C10···H7^vi^	3.2123
C14···C17	2.770 (4)	C10···H10^v^	3.3064
O1···O1^i^	3.100 (5)	C11···H7^i^	3.4762
O1···O2^ii^	3.559 (5)	C11···H18A^iii^	3.4878
O1···C1^i^	3.212 (5)	C11···H18B^viii^	2.8982
O1···C3^ii^	3.533 (5)	C12···H18B^viii^	3.2615
O1···C8^ii^	3.548 (5)	C13···H1^vi^	3.1655
O1···C13^iii^	3.470 (6)	C13···H4^v^	2.6957
O1···C13^iv^	3.260 (5)	C14···H1^vi^	3.0044
O1···C14^iv^	3.543 (6)	C14···H4^v^	2.8629
O2···O1^ii^	3.559 (5)	C14···H5^v^	3.2908
O2···N2^v^	3.012 (4)	C14···H7^iv^	3.5029
O2···C7^ii^	3.533 (6)	C16···H4^x^	2.9759
O2···C10^v^	3.360 (4)	C16···H16^xi^	3.4437
O3···C2^iv^	3.523 (5)	C17···H4^x^	3.2407
O3···C6^i^	3.217 (4)	C17···H5^x^	3.5108
O3···C7^i^	3.111 (4)	C17···H16^xi^	3.3564
O3···C10^iv^	3.415 (6)	C18···H5^xii^	2.8674
O3···C18^iii^	3.579 (5)	C18···H6^xii^	3.2749
N1···N1^iv^	3.304 (5)	C18···H6^iv^	3.5518
N1···N2^iv^	3.513 (6)	H1···O1^i^	2.7414
N2···O2^v^	3.012 (4)	H1···C4^ii^	3.4921
N2···N1^iv^	3.513 (6)	H1···C7^i^	3.3884
C1···O1^i^	3.212 (5)	H1···C9^i^	3.4609
C1···C4^ii^	3.418 (6)	H1···C13^iii^	3.1655
C1···C8^ii^	3.569 (5)	H1···C14^iii^	3.0044
C1···C13^iv^	3.454 (6)	H1···H4^ii^	3.5465
C2···O3^iv^	3.523 (5)	H1···H7^i^	2.8309
C2···C11^iv^	3.357 (5)	H1···H13^iii^	2.7376
C2···C12^iv^	3.579 (6)	H1···H13^iv^	3.3501
C3···O1^ii^	3.533 (5)	H1···H14^iii^	2.4082
C3···C7^ii^	3.548 (6)	H2···O1^vi^	3.5116
C3···C9^ii^	3.335 (5)	H2···O2^v^	2.2147
C4···C1^ii^	3.418 (6)	H2···C3^v^	3.3375
C4···C13^v^	3.461 (5)	H2···H4^v^	3.3991
C4···C14^v^	3.482 (5)	H2···H7^vi^	3.0983
C6···O3^i^	3.217 (4)	H2···H18B^viii^	3.3788
C6···C10^ii^	3.382 (5)	H4···C13^v^	2.6957
C6···C18^iv^	3.531 (6)	H4···C14^v^	2.8629
C7···O2^ii^	3.533 (6)	H4···C16^vii^	2.9759
C7···O3^i^	3.111 (4)	H4···C17^vii^	3.2407
C7···C3^ii^	3.548 (6)	H4···H1^ii^	3.5465
C7···C14^iv^	3.389 (5)	H4···H2^v^	3.3991
C7···C15^iv^	3.565 (6)	H4···H13^v^	2.3807
C8···O1^ii^	3.548 (5)	H4···H14^v^	2.6851
C8···C1^ii^	3.569 (5)	H4···H16^vii^	2.4088
C9···C3^ii^	3.335 (5)	H4···H17^vii^	2.9362
C9···C13^iv^	3.426 (5)	H5···C14^v^	3.2908
C9···C14^iv^	3.356 (6)	H5···C17^vii^	3.5108
C10···O2^v^	3.360 (4)	H5···C18^ix^	2.8674
C10···O3^iv^	3.415 (6)	H5···H14^v^	2.6245
C10···C6^ii^	3.382 (5)	H5···H17^vii^	2.7383
C10···C11^iv^	3.516 (6)	H5···H18A^ix^	2.7813
C11···C2^iv^	3.357 (5)	H5···H18B^ix^	2.7145
C11···C10^iv^	3.516 (6)	H5···H18C^ix^	2.6172
C12···C2^iv^	3.579 (6)	H5···H18C^iv^	3.5212
C13···O1^vi^	3.470 (6)	H6···O2^iii^	3.1915
C13···O1^iv^	3.260 (5)	H6···O3^i^	2.7276
C13···C1^iv^	3.454 (6)	H6···C10^ii^	3.4658
C13···C4^v^	3.461 (5)	H6···C18^ix^	3.2749
C13···C9^iv^	3.426 (5)	H6···C18^iv^	3.5518
C14···O1^iv^	3.543 (6)	H6···H10^iii^	3.5436
C14···C4^v^	3.482 (5)	H6···H10^ii^	3.3155
C14···C7^iv^	3.389 (5)	H6···H18A^iv^	2.9828
C14···C9^iv^	3.356 (6)	H6···H18B^ix^	3.3372
C15···C7^iv^	3.565 (6)	H6···H18C^ix^	2.4385
C18···O3^vi^	3.579 (5)	H6···H18C^iv^	3.3689
C18···C6^iv^	3.531 (6)	H7···O2^ii^	3.4265
O1···H7	2.5030	H7···O3^i^	2.5113
O2···H4	2.6243	H7···N1^i^	3.2406
O2···H10	2.7081	H7···N2^iii^	3.5138
O3···H2	3.0580	H7···C10^iii^	3.2123
O3···H17	2.5031	H7···C11^i^	3.4762
N1···H1	2.3347	H7···C14^iv^	3.5029
N2···H10	2.4367	H7···H1^i^	2.8309
N2···H13	2.6299	H7···H2^iii^	3.0983
C1···H10	3.2867	H7···H10^iii^	2.6721
C3···H1	3.2832	H7···H14^iv^	3.2138
C3···H4	2.6821	H7···H18A^iv^	3.3708
C3···H10	2.7745	H10···O2^v^	2.6112
C4···H6	3.2746	H10···O3^iv^	3.4330
C5···H7	3.2733	H10···C3^v^	3.5196
C6···H4	3.2772	H10···C6^ii^	3.4010
C7···H5	3.2633	H10···C7^vi^	3.2563
C8···H5	3.2697	H10···C10^v^	3.3064
C8···H7	3.2937	H10···H6^vi^	3.5436
C9···H1	3.1796	H10···H6^ii^	3.3155
C9···H4	3.2541	H10···H7^vi^	2.6721
C9···H6	3.2435	H10···H10^v^	2.4774
C10···H1	2.5273	H13···O1^vi^	2.6329
C10···H2	2.4517	H13···O1^iv^	3.1636
C11···H13	2.7298	H13···O2^v^	3.0022
C11···H17	2.6041	H13···C1^vi^	2.9937
C12···H2	2.5663	H13···C1^iv^	3.2392
C12···H14	3.2687	H13···C4^v^	3.0275
C12···H16	3.2653	H13···H1^vi^	2.7376
C13···H2	2.6000	H13···H1^iv^	3.3501
C13···H17	3.2627	H13···H4^v^	2.3807
C14···H16	3.2482	H14···O3^vi^	3.5214
C14···H18A	2.6299	H14···N1^vi^	3.4032
C14···H18B	2.9350	H14···C1^vi^	3.1600
C14···H18C	3.2781	H14···C4^v^	3.0644
C15···H13	3.2778	H14···C5^v^	3.0350
C15···H17	3.2748	H14···C7^iv^	3.3602
C16···H14	3.2489	H14···C9^iv^	3.5858
C16···H18A	3.2627	H14···H1^vi^	2.4082
C16···H18B	2.9920	H14···H4^v^	2.6851
C16···H18C	2.6162	H14···H5^v^	2.6245
C17···H13	3.2631	H14···H7^iv^	3.2138
C18···H14	2.6690	H16···O2^x^	3.4097
C18···H16	2.6813	H16···C4^x^	3.0884
H1···H10	3.4674	H16···C16^xi^	3.4437
H2···H10	2.2688	H16···C17^xi^	3.3564
H2···H13	2.0957	H16···H4^x^	2.4088
H4···H5	2.3230	H16···H16^xi^	3.0510
H5···H6	2.3532	H16···H17^xi^	2.8844
H6···H7	2.3374	H17···C4^x^	3.3186
H13···H14	2.3314	H17···C5^x^	3.2163
H14···H18A	2.4703	H17···H4^x^	2.9362
H14···H18B	3.0000	H17···H5^x^	2.7383
H14···H18C	3.5445	H17···H16^xi^	2.8844
H16···H17	2.3230	H17···H18A^iii^	3.4121
H16···H18A	3.5215	H17···H18B^viii^	3.5420
H16···H18B	3.0907	H17···H18C^xi^	3.5867
H16···H18C	2.4407	H18A···O3^vi^	2.6017
O1···H1^i^	2.7414	H18A···C6^iv^	3.1090
O1···H2^iii^	3.5116	H18A···C7^iv^	3.3403
O1···H13^iii^	2.6329	H18A···C11^vi^	3.4878
O1···H13^iv^	3.1636	H18A···H5^xii^	2.7813
O2···H2^v^	2.2147	H18A···H6^iv^	2.9828
O2···H6^vi^	3.1915	H18A···H7^iv^	3.3708
O2···H7^ii^	3.4265	H18A···H17^vi^	3.4121
O2···H10^v^	2.6112	H18A···H18B^xiii^	3.1100
O2···H13^v^	3.0022	H18B···O3^viii^	3.2105
O2···H16^vii^	3.4097	H18B···N2^viii^	3.1184
O3···H6^i^	2.7276	H18B···C5^xii^	3.4854
O3···H7^i^	2.5113	H18B···C11^viii^	2.8982
O3···H10^iv^	3.4330	H18B···C12^viii^	3.2615
O3···H14^iii^	3.5214	H18B···H2^viii^	3.3788
O3···H18A^iii^	2.6017	H18B···H5^xii^	2.7145
O3···H18B^viii^	3.2105	H18B···H6^xii^	3.3372
N1···H7^i^	3.2406	H18B···H17^viii^	3.5420
N1···H14^iii^	3.4032	H18B···H18A^xiii^	3.1100
N2···H7^vi^	3.5138	H18B···H18B^xiii^	3.2931
N2···H18B^viii^	3.1184	H18C···C5^xii^	3.1753
C1···H13^iii^	2.9937	H18C···C5^iv^	3.4913
C1···H13^iv^	3.2392	H18C···C6^xii^	3.0855
C1···H14^iii^	3.1600	H18C···C6^iv^	3.3918
C3···H2^v^	3.3375	H18C···H5^xii^	2.6172
C3···H10^v^	3.5196	H18C···H5^iv^	3.5212
C4···H1^ii^	3.4921	H18C···H6^xii^	2.4385
C4···H13^v^	3.0275	H18C···H6^iv^	3.3689
C4···H14^v^	3.0644	H18C···H17^xi^	3.5867
C4···H16^vii^	3.0884	H18C···H18C^xiv^	3.5069
C4···H17^vii^	3.3186		
			
C1—O1—C9	117.98 (18)	C16—C15—C18	121.33 (19)
N2—N1—C10	117.43 (19)	C15—C16—C17	121.38 (19)
N1—N2—C11	117.00 (18)	C12—C17—C16	120.3 (3)
O1—C1—C2	125.95 (18)	N1—N2—H2	121.503
C1—C2—C3	119.63 (18)	C11—N2—H2	121.492
C1—C2—C10	118.57 (17)	O1—C1—H1	117.026
C3—C2—C10	121.73 (19)	C2—C1—H1	117.025
O2—C3—C2	123.18 (18)	C5—C4—H4	119.839
O2—C3—C8	123.00 (17)	C8—C4—H4	119.837
C2—C3—C8	113.82 (19)	C4—C5—H5	119.690
C5—C4—C8	120.3 (2)	C6—C5—H5	119.685
C4—C5—C6	120.63 (19)	C5—C6—H6	120.064
C5—C6—C7	119.87 (19)	C7—C6—H6	120.067
C6—C7—C9	118.7 (2)	C6—C7—H7	120.626
C3—C8—C4	121.51 (19)	C9—C7—H7	120.626
C3—C8—C9	120.80 (17)	N1—C10—H10	121.301
C4—C8—C9	117.68 (18)	C2—C10—H10	121.295
O1—C9—C7	115.60 (19)	C12—C13—H13	119.863
O1—C9—C8	121.67 (17)	C14—C13—H13	119.863
C7—C9—C8	122.74 (18)	C13—C14—H14	119.442
N1—C10—C2	117.4 (2)	C15—C14—H14	119.438
O3—C11—N2	122.18 (18)	C15—C16—H16	119.316
O3—C11—C12	121.41 (18)	C17—C16—H16	119.306
N2—C11—C12	116.42 (19)	C12—C17—H17	119.868
C11—C12—C13	123.77 (18)	C16—C17—H17	119.869
C11—C12—C17	117.3 (2)	C15—C18—H18A	109.473
C13—C12—C17	118.88 (18)	C15—C18—H18B	109.470
C12—C13—C14	120.27 (19)	C15—C18—H18C	109.469
C13—C14—C15	121.1 (2)	H18A—C18—H18B	109.473
C14—C15—C16	118.08 (18)	H18A—C18—H18C	109.474
C14—C15—C18	120.6 (2)	H18B—C18—H18C	109.468
			
C1—O1—C9—C7	−178.84 (15)	H6—C6—C7—H7	−1.1
C1—O1—C9—C8	1.0 (3)	C6—C7—C9—O1	179.93 (17)
C9—O1—C1—C2	−1.2 (3)	C6—C7—C9—C8	0.1 (3)
C9—O1—C1—H1	178.8	H7—C7—C9—O1	−0.1
N2—N1—C10—C2	177.37 (15)	H7—C7—C9—C8	−179.9
N2—N1—C10—H10	−2.6	C3—C8—C9—O1	2.0 (3)
C10—N1—N2—C11	−179.93 (16)	C3—C8—C9—C7	−178.21 (16)
C10—N1—N2—H2	0.1	C4—C8—C9—O1	−178.83 (16)
N1—N2—C11—O3	0.6 (3)	C4—C8—C9—C7	1.0 (3)
N1—N2—C11—C12	−179.89 (15)	O3—C11—C12—C13	−160.68 (18)
H2—N2—C11—O3	−179.4	O3—C11—C12—C17	16.1 (3)
H2—N2—C11—C12	0.1	N2—C11—C12—C13	19.8 (3)
O1—C1—C2—C3	−1.5 (3)	N2—C11—C12—C17	−163.39 (16)
O1—C1—C2—C10	−178.67 (16)	C11—C12—C13—C14	177.51 (16)
H1—C1—C2—C3	178.5	C11—C12—C13—H13	−2.5
H1—C1—C2—C10	1.3	C11—C12—C17—C16	−178.00 (16)
C1—C2—C3—O2	−175.92 (17)	C11—C12—C17—H17	2.0
C1—C2—C3—C8	4.1 (3)	C13—C12—C17—C16	−1.1 (3)
C1—C2—C10—N1	−8.1 (3)	C13—C12—C17—H17	178.9
C1—C2—C10—H10	171.9	C17—C12—C13—C14	0.8 (3)
C3—C2—C10—N1	174.81 (16)	C17—C12—C13—H13	−179.2
C3—C2—C10—H10	−5.2	C12—C13—C14—C15	0.1 (3)
C10—C2—C3—O2	1.1 (3)	C12—C13—C14—H14	−179.9
C10—C2—C3—C8	−178.81 (15)	H13—C13—C14—C15	−179.9
O2—C3—C8—C4	−3.5 (3)	H13—C13—C14—H14	0.1
O2—C3—C8—C9	175.66 (17)	C13—C14—C15—C16	−0.7 (3)
C2—C3—C8—C4	176.47 (15)	C13—C14—C15—C18	179.17 (17)
C2—C3—C8—C9	−4.4 (3)	H14—C14—C15—C16	179.3
C5—C4—C8—C3	178.06 (17)	H14—C14—C15—C18	−0.8
C5—C4—C8—C9	−1.1 (3)	C14—C15—C16—C17	0.4 (3)
C8—C4—C5—C6	0.2 (3)	C14—C15—C16—H16	−179.6
C8—C4—C5—H5	−179.8	C14—C15—C18—H18A	33.7
H4—C4—C5—C6	−179.8	C14—C15—C18—H18B	−86.3
H4—C4—C5—H5	0.2	C14—C15—C18—H18C	153.7
H4—C4—C8—C3	−1.9	C16—C15—C18—H18A	−146.4
H4—C4—C8—C9	178.9	C16—C15—C18—H18B	93.6
C4—C5—C6—C7	0.9 (3)	C16—C15—C18—H18C	−26.4
C4—C5—C6—H6	−179.1	C18—C15—C16—C17	−179.45 (17)
H5—C5—C6—C7	−179.1	C18—C15—C16—H16	0.5
H5—C5—C6—H6	0.9	C15—C16—C17—C12	0.5 (3)
C5—C6—C7—C9	−1.1 (3)	C15—C16—C17—H17	−179.5
C5—C6—C7—H7	178.9	H16—C16—C17—C12	−179.5
H6—C6—C7—C9	178.9	H16—C16—C17—H17	0.5

Symmetry codes: (i) −*x*+1, −*y*+1, −*z*+2; (ii) −*x*+2, −*y*+1, −*z*+2; (iii) *x*, *y*+1, *z*; (iv) −*x*+1, −*y*, −*z*+2; (v) −*x*+2, −*y*, −*z*+2; (vi) *x*, *y*−1, *z*; (vii) *x*+1, *y*+1, *z*+1; (viii) −*x*+1, −*y*−1, −*z*+1; (ix) *x*+1, *y*+2, *z*+1; (x) *x*−1, *y*−1, *z*−1; (xi) −*x*, −*y*−1, −*z*+1; (xii) *x*−1, *y*−2, *z*−1; (xiii) −*x*+1, −*y*−2, −*z*+1; (xiv) −*x*, −*y*−2, −*z*+1.

### Hydrogen-bond geometry (Å, º)

**Table:**

*D*—H···*A*	*D*—H	H···*A*	*D*···*A*	*D*—H···*A*
N2—H2···O2^v^	0.88	2.22	3.012 (4)	151

Symmetry code: (v) −*x*+2, −*y*, −*z*+2.
